# Supplementation of coated sodium butyrate relieved weaning stress and reshaped microbial flora in weaned lambs

**DOI:** 10.3389/fvets.2024.1423920

**Published:** 2024-07-22

**Authors:** Fangfang Zhao, Wenhao He, Tianyou Wu, Mawada Elmhadi, Ning Jiang, Aizhong Zhang, Pengyu Guan

**Affiliations:** ^1^College of Animal Science and Veterinary Medicine, Heilongjiang Bayi Agricultural University, Daqing, China; ^2^Heilongjiang Provincial Key Laboratory of Efficient Utilization of Feed Resources and Nutrition Manipulation in Cold Region, Daqing, China; ^3^Bright Farming Co., Ltd., Shanghai, China; ^4^Laboratory of Metabolic Manipulation of Herbivorous Animal Nutrition, College of Animal Science and Technology, Yangzhou University, Yangzhou, China

**Keywords:** coated sodium butyrate, weaned lamb, growth performance, antioxidant capacity, gut microbiota

## Abstract

Weaning is an important period in the growth and development of lambs. Thus, effectively reducing the occurrence of weaning stress is critical for maintaining lamb production. Coated sodium butyrate has been shown to reduce inflammation, promote intestinal health, and maintain homeostasis. However, the application and potential mechanism of coated sodium butyrate in alleviating weaning stress in lambs are still unclear. To evaluate the effects of coated sodium butyrate on the growth performance, antioxidant capacity, and gut microbiota of weaned lambs, 10 weaned lambs of 21-day-old were randomly divided into two groups: the CON group (basal diet) and the NaB group (basal diet +3 g/kg of coated sodium butyrate). The trial lasted 21 days. The experimental results showed that compared to the CON group, coated sodium butyrate supplementation in the diet significantly increased the average daily weight gain and daily feed intake of lambs (*p* < 0.05). In addition, compared to the CON group, the addition of coated sodium butyrate also significantly decreased the serum MDA level of lambs (*p* < 0.05). Notably, the addition of coated sodium butyrate did not have a significant effect on the cecal microbiota, while increasing the diversity of colonic microbiota and promoting the abundance of *Lachnospiraceae*, *Verrucomicrobiota*, *Akkermansia*, *Roseburia*, and *Sinobacteraceae*, which are associated with the nutrient absorption of lambs (*p* < 0.05). These results indicate that dietary supplementation with coated sodium butyrate could promote the growth and antioxidant capacity of weaned lambs and alleviate weaning stress.

## 1 Introduction

In sheep production, to meet the needs of intensive production, artificial weaning interventions are usually carried out on suckling lambs to improve reproductive performance, shorten intergenerational intervals, and reduce feed costs (1). Emerging evidence indicates that the choice of different weaning methods (complete separation weaning and stage separation weaning) will affect the growth performance of lambs (2–4). Thus, early-weaning schema selection determines the quality of lambs. Unfortunately, the stress response caused by early weaning in lambs is common (1). On the one hand, the rapid transformation of dietary structure and community relationships as stressors can affect the feeding behavior and physiological function of weaned lambs to varying degrees (5). Moreover, weaning leads to a change in serum cortisol levels in lambs, which is considered an indicator of stress levels (6). Notably, early weaning also caused concerns about production performance and animal welfare (5). Thus, developing strategies to minimize the weaning stress of suckling lambs is important.

The function of sodium butyrate in immune metabolism regulation has been confirmed (7–10). Early weaning has been associated with intestinal barrier dysfunction and immune dysregulation (11). Several studies have confirmed that sodium butyrate can alleviate epithelial damage caused by weaning stress (12). In addition, supplementation of sodium butyrate in the diet improved the intestinal development of suckling lambs (13). From a mechanistic perspective, previous studies indicated that the effect of sodium butyrate may be related to the regulation of inflammatory pathways (14). It is interesting to note that the weaning period is associated with the development of oxidative stress damage and mitochondrial dysfunction (15). Emerging evidence indicates that sodium butyrate participates in the repair of mitochondrial oxidative stress damage (16). Among them, the AMPK-mitophagy pathway may exert a key role (12). Considering the effectiveness of sodium butyrate in alleviating oxidative stress damage, feeding sodium butyrate during the weaning stage seems advantageous. However, the report on the effect of sodium butyrate addition on early-weaned lambs is not comprehensive. Notably, the change in the gut microbiota is associated with mitochondrial damage (17). Emerging evidence suggests that early weaning reduces the abundance and diversity of the gut microbiota (18). Furthermore, the supplementation of butyrate in the diet improved the structural composition of the intestinal microflora of early-weaning animals (19, 20). Thus, the contribution of sodium butyrate to maintaining the homeostasis of the gut microbiota in early-weaning lambs still needs further investigation.

Uncoated sodium butyrate is absorbed immediately in the anterior section of the alimentary canal before it can reach the distal intestine (21), therefore limiting the efficiency of sodium butyrate throughout the gastrointestinal tract (22). Thus, this study aimed to evaluate the function of coated sodium butyrate supplements in the diet of early-weaned lambs on growth performance, antioxidant capacity, and the cecum and colon microbiota changes. The results indicated that 3 g/kg of coated sodium butyrate promoted growth and antioxidant capacity and remodeled the colonic microbiota of weaned lambs. The research results could guide the application of sodium butyrate in early-weaned lambs.

## 2 Materials and methods

### 2.1 Animals, diets, and experimental design

A total of 10 healthy weaned male lambs (South African Meat Merino × small-tailed Han sheep) at the age of 21 days with mean body weight (BW) of 6.53 ± 0.17 kg were randomly divided into two groups with five replicates in each group: the CON group, basal diet without coated sodium butyrate supplementation; and the NaB group, basal diet with 3 g/kg of coated sodium butyrate supplementation. Coated sodium butyrate was purchased from Dongying Herunde Biological Technology Co., Ltd. Sodium butyrate content was 30%, coated mainly with fat to control the release of sodium butyrate. The whole trial lasted for 21 days. During the trial period, all weaned lambs were individually housed in slatted wooden floor pens with a drinking bowl and feed chute. The diets were offered in equal amounts twice a day at 08:00 and 18:00 and allowed *ad libitum* access to feed and water. The feed formulation and chemical composition are shown in Table 1.

**Table 1:** Feed formulation and chemical composition (DM basis, %).

Items	Basal diet
Ingredients (%)	
Alfalfa	7.00
Oat hay	5.00
Corn	45.00
Soybean meal	20.50
Wheat bran	8.00
Corn germ meal	10.00
Ground limestone	2.00
CaHPO_4_	0.50
Salt	0.50
NaHCO_3_	0.50
Premix^1^	1.00
Total	100.00
Nutrient levels^2^	
DM (%)	89.1
DE (MJ/kg)	13.87
CP (%)	21.52
NDF (%)	18.93
ADF (%)	8.21
Calcium (%)	0.86
Phosphorus (%)	0.59

1 The premix provided nutrients per kg of diet as follows: Fe, 25 mg; Zn, 40 mg; Cu, 8 mg; Mn, 40 mg; I, 0.3 mg; Se, 0.2 mg; Co, 0.1 mg; vitamin A, 940 IU; and vitamin E, 20 IU.

2 DE was a calculated value, while others were measured values. DM, dry matter; DE, digestive energy; CP, crude protein; NDF, neutral detergent fiber; ADF, acid detergent fiber.

The chemical composition of the feed consumed by the lambs was assessed for dry matter and total *N*, according to the Association of Official Analytical Chemists (23). The crude protein content in the feed was calculated as the total *N* × 6.25. The determination of neutral detergent fiber contents was conducted according to the procedure of Van Soest (24), and acid detergent fiber was determined by procedures described by Goering and Van Soest (25). Calcium and phosphorus were quantified using inductively coupled plasma emission spectroscopy after acid digestion. The instrument used for this purpose was the Atom Scan 25 Plasma Spectroscopy, manufactured by Thermo Jarrell Ash Corp. and located in Grand Junction, Colorado.

### 2.2 Growth performance measurement

All weaned lambs fasted for 12 h before the start and the end of the trial, respectively. Subsequently, BW was recorded as the initial and final weight, and the average daily gain (ADG) was calculated. The recovery of surplus feed was carried out 1 h after the end of feeding daily, and then the average daily feed intake (ADFI) was recorded.

### 2.3 Blood sample collection and analysis

At the end of the trial, 10 mL of blood samples of weaned lambs were collected from the jugular vein before morning feed. Then the blood was centrifuged at 3,000×*g* for 10 min at room temperature to obtain the serum samples. The separated serum was stored at −80°C for future analyses.

Serum antioxidant indicators, such as total antioxidant capacity (T-AOC), malondialdehyde (MDA), total superoxide dismutase (T-SOD), catalase (CAT), and glutathione peroxidase (GSH-Px), were measured by commercial kits (Jiancheng Bioengineering Institute, Nanjing, China) according to the manufacturer’s instructions and protocol.

### 2.4 The collection of cecal and colonic contents

All weaned lambs were euthanized after blood samples were collected. The cecal and colonic contents were then collected by stripping off the ceca and colon, and immediately transferred into 2 mL sterilized tubes for fast freezing in liquid nitrogen. Finally, the samples of cecal and colonic contents were stored at −80°C for microbiota analysis.

### 2.5 Microbiota analysis

Microbial DNA in the cecal and colonic contents was extracted using the OMEGA Soil DNA Kit (Omega Bio-tek, Norcross, GA, USA) according to the manufacturer’s protocol. Then, the V3–V4 region of the bacterial 16S rRNA gene was amplified using the forward primer 338F (5′-ACTCCTACGGGAGGCAGCA-3′) and the reverse primer 806R (5′-GGACTACHVGGGTWTCTAAT-3′). All PCR reactions were carried out with 15 μL of Phusion® High-Fidelity PCR Master Mix (New England Biolabs), 0.2 μM of forward and reverse primers, and approximately 10 ng of template DNA. Thermal cycling consisted of initial denaturation at 98°C for 1 min, followed by 30 cycles of denaturation at 98°C for 10 s, annealing at 50°C for 30 s, and elongation at 72°C for 30 s and 72°C for 5 min. The resultant PCR products were extracted from a 2% agarose gel and further purified using a Universal DNA Purification Kit (TianGen, China). After the amplification and purification of PCR products, amplicons were sequenced with 250 bp paired-end reads using the Illumina NovaSeq platform.

### 2.6 Statistical analysis

Distributed data and non-normally distributed data were subjected to the student’s t-test and Wilcoxon rank-sum test using SPSS 22.0 (IBM, USA), respectively. All data were expressed as mean ± SD. A *p*-value of <0.05 indicated significant differences. The data were visualized using GraphPad Prism (GraphPad Software, Ver. 9.0, CA, USA) and ggplot2 in R language (4.0.3).

## 3 Results

### 3.1 Coated sodium butyrate promoted the growth of weaned lambs

To evaluate the effect of coated sodium butyrate supplementation on the growth performance of weaned lamb, changes in BW and feed intake were recorded during the whole trial. As shown in Table 2, compared with the CON group, coated sodium butyrate significantly increased the ADFI and ADG of weaned lambs (*p* < 0.05). Consistent with expectations, the initial weight of weaned lambs had no difference (*p* > 0.05). Furthermore, the diet supplemented with coated sodium butyrate increased the final weight of weaned lambs compared to the CON group.

**Table 2:** Effect of coated sodium butyrate supplement on growth performance in weaned lambs (*n* = 5).

Item^1^	Treatment		*p*-value
	CON	NaB	
Initial weight, kg	6.42 ± 0.42	6.64 ± 0.65	0.534
Final weight, kg	8.98 ± 0.67	9.66 ± 0.40	0.088
ADFI, g/d	329.21 ± 14.60	361.14 ± 22.54	0.029
ADG, g/d	121.90 ± 16.36	159.05 ± 23.71	0.020

1 Effect of coated sodium butyrate supplement on growth performance in weaned lambs (*n* = 5).

### 3.2 Coated sodium butyrate improved the serum antioxidant indicator

The changes in serum antioxidant indicators of weaned lambs were shown in two groups, and the results are shown in Table 3. Compared to the CON group, the diet supplemented with coated sodium butyrate had no effect on the serum antioxidant indicators of T-AOC, T-SOD, GSH-Px, and CAT (*p* > 0.05), while the NaB group showed significantly decreased serum MDA level.

**Table 3:** Effect of coated sodium butyrate supplement on the serum antioxidant indicators in weaned lambs (*n* = 5).

Item^1^	Treatment		*p*-value
	CON	NaB	
T-AOC, U/mL	0.90 ± 0.07	0.93 ± 0.05	0.394
T-SOD, U/mL	70.89 ± 7.90	67.73 ± 4.66	0.463
GSH-Px, U/mL	146.27 ± 22.16	143.40 ± 9.20	0.301
CAT, U/mL	5.17 ± 0.41	5.24 ± 0.52	0.807
MDA, nmol/mL	5.35 ± 0.97	4.21 ± 0.16	0.031

1 Effect of coated sodium butyrate supplement on the serum antioxidant indicators in weaned lambs (*n* = 5).

### 3.3 Coated sodium butyrate had no effect on cecal microbiota

**Figure 1:**
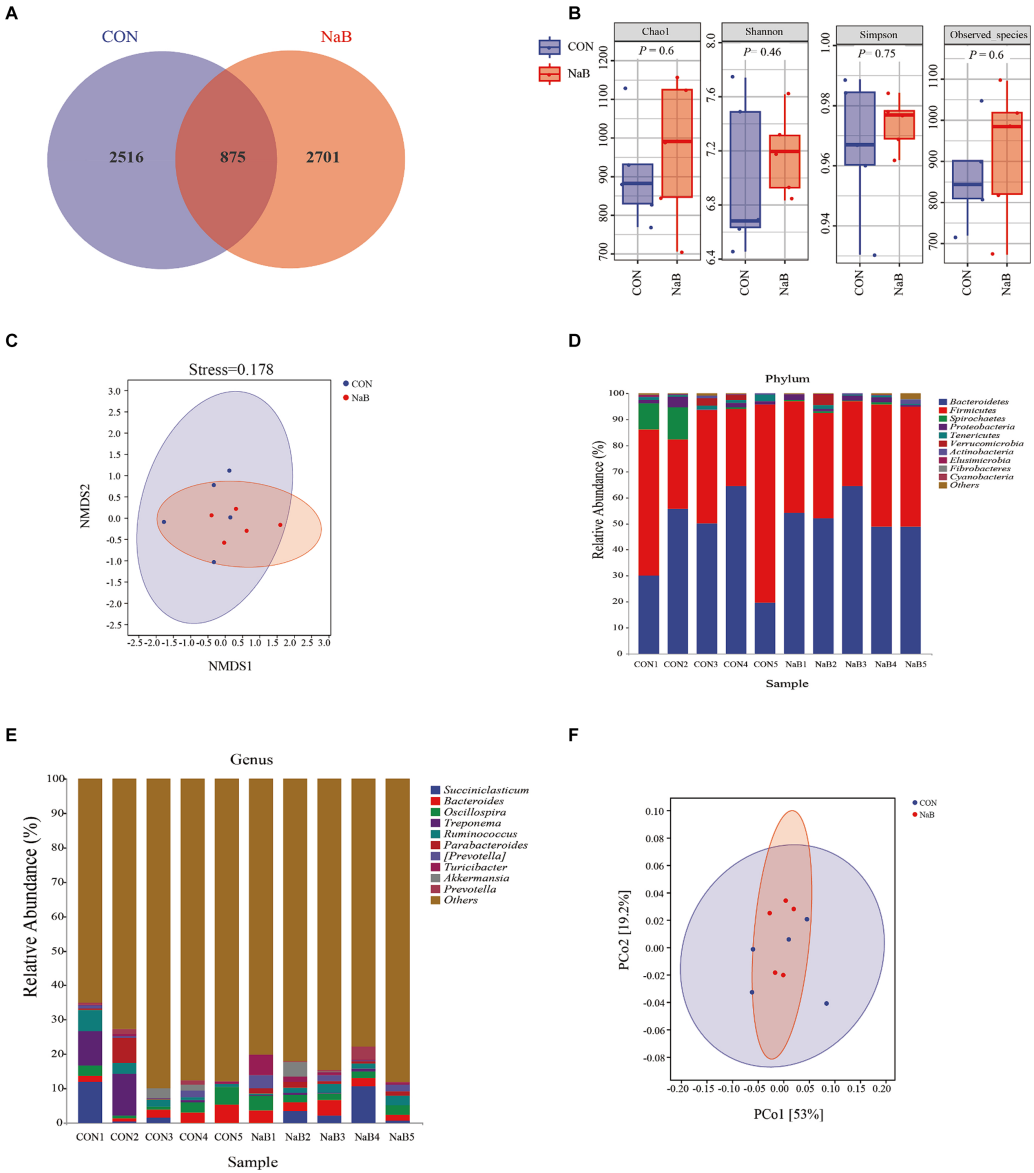
Effect of the diet supplemented with coated sodium butyrate on the cecal microbiota of weaned lambs (*N* = 5). **(A)** Venn diagram of bacterial OTUs in the cecum. **(B)** The α-diversity of the cecal microbiota. **(C)** The β-diversity of the cecal microbiota proceeded by NMDS analysis under the unweighted_unifrac method. **(D)** A comparison of phylum-level proportional abundance of the cecum between the CON group and the NaB group. **(E)** A comparison of genus-level proportional abundance of the cecum between the CON group and the NaB group. **(F)** The functional prediction of cecal microbiota proceeded by PCoA analysis under PICRUSt2 with the unweighted_unifrac method.

To investigate whether coated sodium butyrate supplementation reshaped cecal microbiota, 16S rDNA gene sequencing was performed using cecal contents of weaned lambs. In total, 448,487 and 449,146 raw reads were obtained for the CON and NaB groups, respectively. Furthermore, 357,579 and 369,090 clean reads for the CON and NaB groups were obtained through quality control and filtering. As shown in Figure 1A, a total of 875 shared OTUs were observed between the CON group and the NaB group. In addition, the unique OTU features of the CON group and the NaB group were 2,516 and 2,701, respectively. The α-diversity of cecal microbiota is shown in Figure 1B. The diet supplemented with coated sodium butyrate in lambs had no effect on the richness of cecal microbiota than the CON group (*p* > 0.05). In addition, as shown in Figure 1C, the β-diversity (NMDS, based on unweighted_unifrac) of the cecal microbiota also showed no change between the NaB group and the CON group (*p* > 0.05). The relative abundance of cecal microbiota in each phylum is shown in Figure 1D.

*Bacteroidetes* and *Firmicutes* were the dominant phyla in the two groups. However, at the genus level, a large number of unannotatable microbial classifications were observed. In addition, *Succiniclasticum*, *Bacteroides*, and *Oscillospira* were the predominant microflora in the annotatable microbiota (Figure 1E). To further investigate the potential functions of microbiota in two groups, the PICRUSt2 analysis was carried out. As shown in Figure 1F, the functional unit of PCoA analysis (based on Bray–Curtis) also indicated that the microbial community of cecal contents in the two groups was similar.

### 3.4 Coated sodium butyrate reshaped the colonic microbial flora

**Figure 2:**
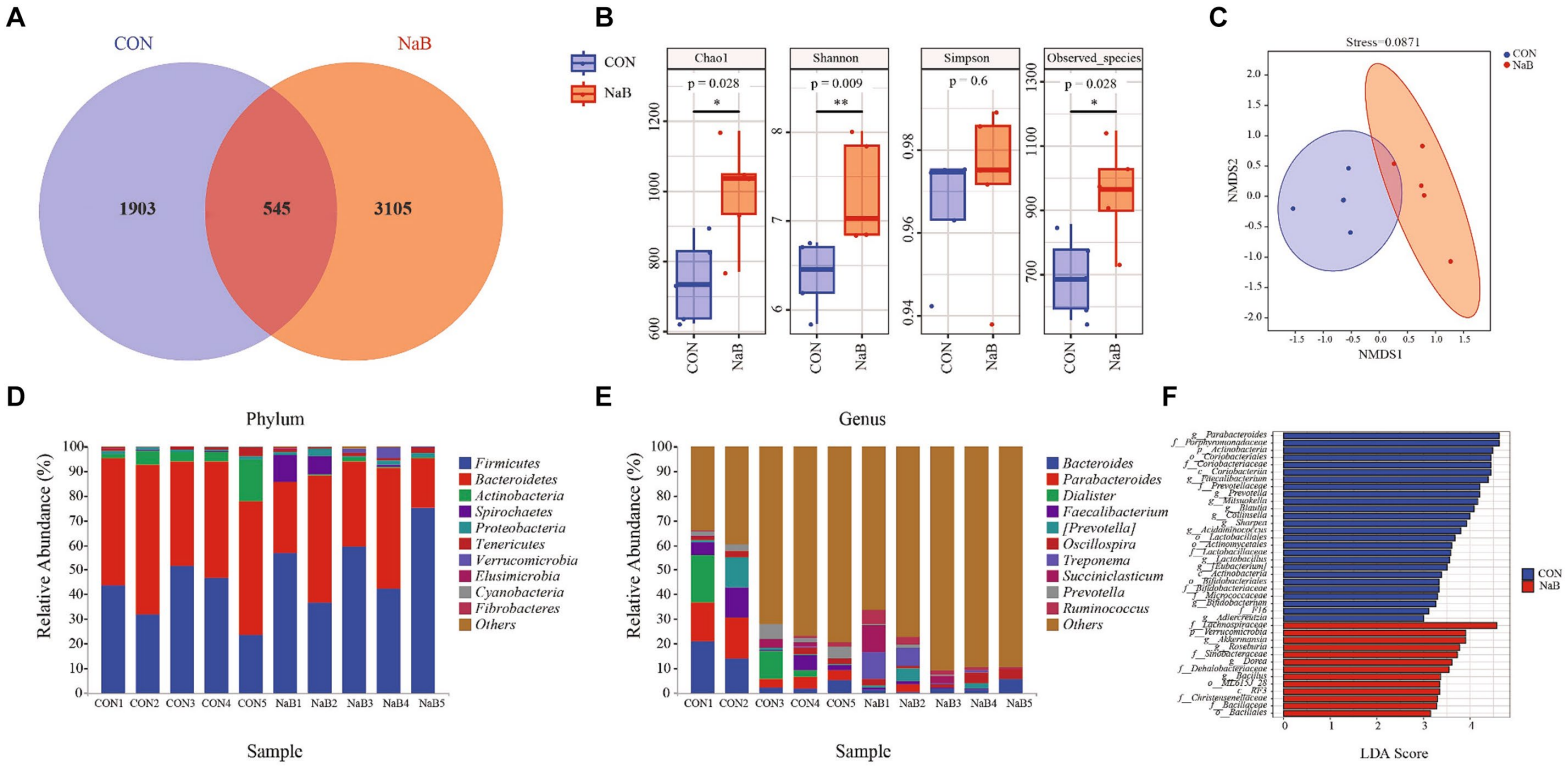
Effect of the diet supplemented with coated sodium butyrate on colonic microbiota of weaned lambs (*N* = 5). **(A)** Venn diagram of bacterial OTUs in the colon. **(B)** The α-diversity of colonic microbiota. **(C)** The β-diversity of colonic microbiota proceeded by NMDS analysis under the unweighted_unifrac method. **(D)** A comparison of the phylum-level proportional abundance of the cecum between the CON group and the NaB group. **(E)** A comparison of the genus-level proportional abundance of the cecum between the CON group and the NaB group. **(F)** The LDA score of colonic microbiota between the CON and NaB groups.

**Figure 3:**
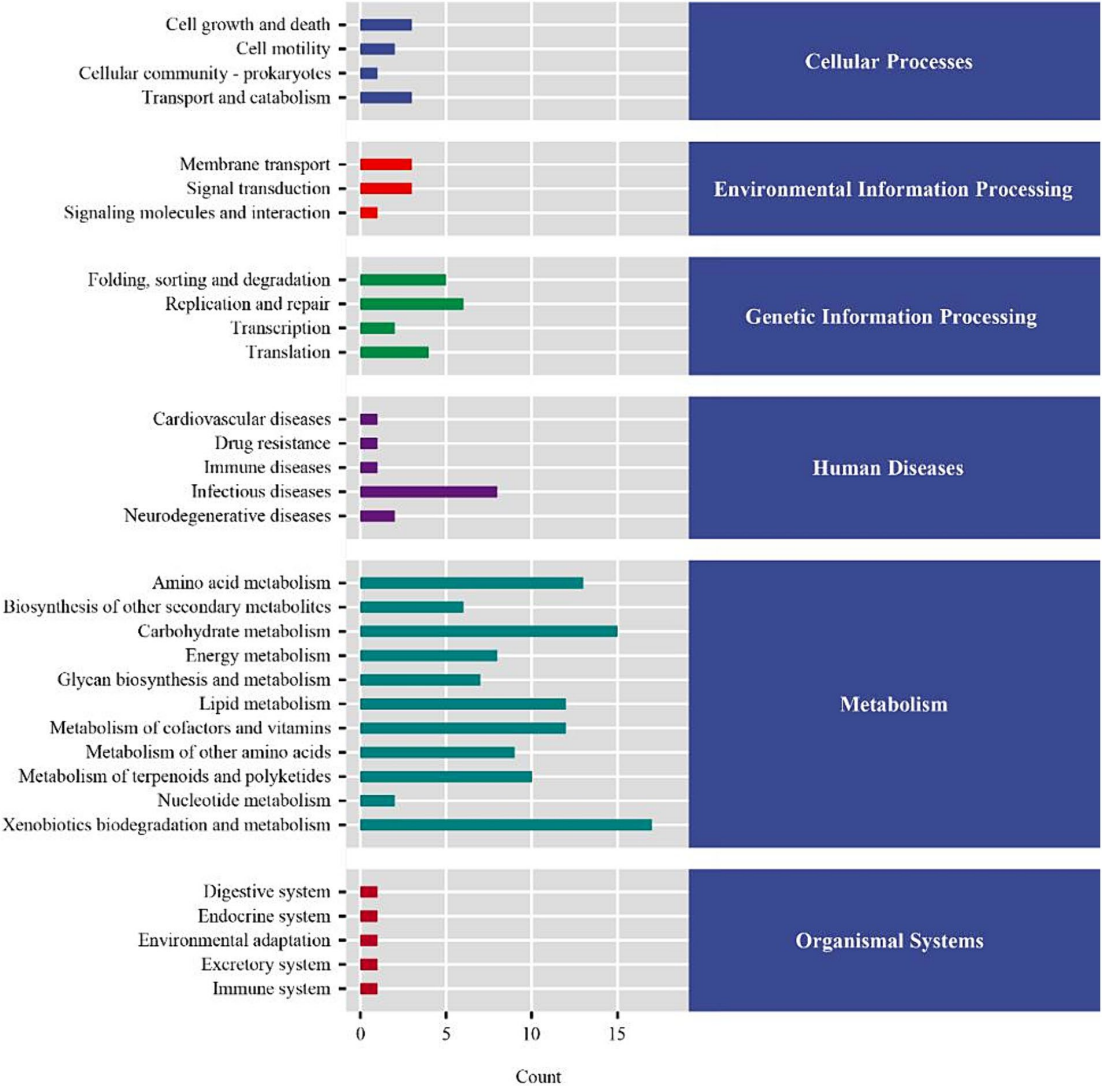
KEGG enrichment analysis of the difference in colonic microbial flora between the CON and NaB groups (*N* = 5).

To further explore the effect of the diet supplemented with coated sodium butyrate on colonic microbiota change, the microbial flora of the colon was measured. In total, 456,776 and 481,231 raw reads were obtained for the CON and NaB groups, respectively. The Venn diagram of colonic microbiota shown in Figure 2A indicates that the CON group had 1,903 unique OTU features, the NaB group had 3,105 unique features, and 545 shared OTU features between the two groups. As shown in Figure 2B, compared to the CON group, the NaB group significantly improved Chao1, Observed species, and Shannon indices (*p* < 0.05). The β-diversity was evaluated to reflect the similarity of colonic microbiota between the CON and NaB groups. The results of the NMDS analysis in Figure 2C indicate a significant separation between the CON and NaB groups (*p* < 0.05). At the phylum level, *Firmicutes*, *Bacteroidetes*, and *Actinobacteria* were predominant (Figure 2D). At the genus level, *Bacteroides*, *Parabacteroides*, and *Dialister* were the predominant colonic microflora (Figure 2E). To further explore the specific bacteria associated with the CON and NaB groups, LEfSe and LDA analyses were performed. As shown in Figure 2F, there were 39 taxa of biomarkers between the two groups with an LDA score of >3 and a *p*-value of <0.05. Compared to the CON group, the NaB group significantly improved the relative abundances of *Lachnospiraceae*, *Verrucomicrobia*, *Akkermansia*, *Roseburia*, and *Sinobacteraceae*. Next, the predicted results of differential microbial community function based on the KEGG database are shown in Figure 3. The KEGG enrichment analysis of the difference in colonic microbial flora showed that 32 pathways were identified significantly (*p* < 0.05) between the CON and NaB groups. Further analysis revealed that 11 of 32 pathways were related to metabolism. Specifically, the main metabolic pathways, such as amino acid metabolism, carbohydrate metabolism, lipid metabolism, metabolism of cofactors and vitamins, xenobiotic biodegradation and metabolism, had changed after the supplementation of coated sodium butyrate to weaned lambs. This result indicated that coated sodium butyrate supplementation induced colonic microbial flora reshaping, which might be involved in the metabolic capacity of weaned lambs.

## 4 Discussion

Weaning is a necessary stage in the growth process of lambs, but this stage is often accompanied by a stressful response (26). Weaning-induced stress is typically caused by the rapid dietary change in lambs from breast milk dependence to solid food, social isolation resulting from separation from ewes, and the change in living environment due to weaning (27). Weaning stress affects lambs in various ways, particularly in terms of growth performance and systemic immunity. Several studies have demonstrated that weaning stress decreases the feed consumption of lambs and reduces their growth performance (27). Additionally, weaning stress elevates the cortisol levels in lambs, which can impact lamb immunity (28).

Sodium butyrate has been widely used to improve intestinal health, enhance immunity, and improve animal performance. Several studies have demonstrated that sodium butyrate supplementation improved the carcass traits, growth performance, and intestinal health of finishing pigs (29). In addition, coated sodium butyrate has been proven to improve the intestinal health of laying hens (30). Moreover, coated sodium butyrate could alleviate LPS-induced intestinal inflammation (14). Emerging evidence indicated that sodium butyrate supplementation reduced the colonization of harmful bacteria in the intestinal tract, promoted the homeostasis of intestinal health, and enhanced the growth performance of lambs (13). In addition, sodium butyrate can improve lamb growth performance by enhancing intestinal health and promoting nutrient absorption and utilization (13). From a mechanical perspective, sodium butyrate promotes the proliferation of intestinal epithelial cells, improving the growth performance of animals by enhancing the intestinal barrier (31). Similar to previous research, in our trial, the results also showed that coated sodium butyrate significantly enhanced the ADFI and ADG of weaned lambs, which indicate that sodium butyrate plays a critical role in promoting the growth of weaned lambs. Notably, supplements with coated sodium butyrate also increased the antioxidant capacity in serum (32). Unfortunately, in our trial, coated sodium butyrate did not improve the levels of T-AOC, T-SOD, GSH-Px, and CAT in serum, while the serum MDA level was decreased. This result indicated that coated sodium butyrate might have a limited effect on the antioxidant enzyme system in lambs. Interestingly, the reduction in serum MDA suggested that sodium butyrate was effective in improving lipid peroxidation and reducing oxidative stress (33, 34). In fact, MDA is an important indicator for measuring cell damage caused by the accumulation of oxygen-free radicals. Although the ability of lambs to scavenge oxygen-free radicals did not change after coated sodium butyrate treatment, the plasma membrane damage caused by early weaning still exists. Thus, coated sodium butyrate supplementation might be a potential nutritional strategy to relieve weaning stress induced by the impaired growth performance and immunocompromise of weaned lambs.

Except for the serum antioxidant indicators, coated sodium butyrate regulated the gastrointestinal microbiota of weaned lambs. Previous research has shown that sodium butyrate can alleviate DSS-induced colitis by remodeling gut microbes (35). Emerging evidence indicated that weaning stress in lambs leads to changes in the abundance and diversity of their gastrointestinal microorganisms (36, 37). In this study, it was observed that coated sodium butyrate did not affect the microbiota of the cecum. In fact, the intestinal microorganisms of lambs were still colonizing and developing during the early weaning period (38). In addition, cecal microbiota is mainly related to fermented fiber diets, and the low fiber content in lamb diets may also be an important reason for the unchanged microbiota. Thus, this lack of the effect of coated sodium butyrate may be masked by the development of the cecum and changing microflora due to the incomplete colonization of the cecum by microorganisms. Unlike cecal microbiota, *Firmicutes* and *Bacteroidetes* are the main microbial communities in the colon. It was reported that *Firmicutes* may improve the intestinal barrier to influence host intestinal health and antioxidant capacity (39). Furthermore, *Bacteroidetes* was also associated with promoting host intestinal health by facilitating glycan breakdown in the gut (40). Thus, it is important to study the effect of sodium butyrate on microbial flora due to changes in the colon microbiota. In our trial, coated sodium butyrate increased the relative abundance of several beneficial bacteria in the colon, including *Lachnospiraceae*, *Verrucomicrobia*, *Akkermansia*, *Roseburia*, and *Sinobacteraceae*, which are closely associated with the intestinal barrier, nutrient absorption, and the ability of the colon to absorb nutrients (40). Specifically, *Akkermansia* has been recognized as a model for the next generation of beneficial bacteria, which is essential for maintaining intestinal health and immune system function (41). Previous research has found that *Akkermansia* improved the intestinal barrier function, facilitated the intestinal absorption of nutrients, influenced the intestinal environment, promoted the growth of other beneficial bacteria, and enhanced the host’s immune system (42). In addition, *Lachnospiraceae* also reported improving the intestinal barrier function caused by obesity (43). Several studies indicated that the changes in the relative abundance of *Verrucomicrobia* are related to carbohydrate metabolism (44). It is interesting to note that *Roseburia* can activate the immune response of the host by producing butyric acid (45). In addition, emerging evidence showed that *Sinobacteraceae* is also associated with host immune response (46). Thus, these findings suggested that although coated sodium butyrate may be influenced by various factors, it can target and regulate important microbial groups, which may indirectly enhance lamb health and growth.

## 5 Conclusion

In summary, coated sodium butyrate improved the growth performance and health of weaned lambs through multiple mechanisms. Although there were limited direct effects on certain biomarkers and microbial communities, coated sodium butyrate was effective in promoting improvements in key growth indicators and alleviating oxidative stress. Additionally, it improved the intestinal health of lambs by modulating specific beneficial microbial communities. These findings highlight the potential application of sodium butyrate in sheep production and provide new directions for future research.

## Data availability statement

The datasets presented in this study can be found in online repositories. The names of the repository/repositories and accession number(s) can be found below: https://www.ncbi.nlm.nih.gov/, PRJNA1091061 and PRJNA1091047.

## Ethics statement

All animal experiments adhered to the animal experiment policy of the Animal Care Institution and Ethics Committee of Heilongjiang Bayi Agricultural University (Daqing, China; Grant number, DWKJXY2022019). The study was conducted in accordance with the local legislation and institutional requirements.

## Author contributions

FZ: Conceptualization, Formal analysis, Writing – original draft. WH: Investigation, Methodology, Writing – review & editing. TW: Writing – review & editing. ME: Writing – review & editing. NJ: Writing – review & editing. AZ: Writing – review & editing. PG: Investigation, Writing – review & editing.
